# SILEX: a fast and inexpensive high-quality DNA extraction method suitable for multiple sequencing platforms and recalcitrant plant species

**DOI:** 10.1186/s13007-020-00652-y

**Published:** 2020-08-10

**Authors:** Santiago Vilanova, David Alonso, Pietro Gramazio, Mariola Plazas, Edgar García-Fortea, Paola Ferrante, Maximilian Schmidt, María José Díez, Björn Usadel, Giovanni Giuliano, Jaime Prohens

**Affiliations:** 1 Instituto de Conservación y Mejora de la Agrodiversidad Valenciana, Universitat Politècnica de València, Camino de Vera 14, 46022 Valencia, Spain; 2 Faculty of Life and Environmental Sciences, University of Tsukuba, 1-1-1 Tennodai, 305-8572 Tsukuba, Japan; 3 ENEA, Italian National Agency for New Technologies, Energy and Sustainable Economic Development, Rome, Italy; 4 BG-4 Bioinformatics, Forschungszentrum Jülich, 52428 Jülich, Germany; 5 CEPLAS, Institute for Biological Data Science, Heinrich Heine University Düsseldorf, 40225 Düsselforf, Germany

**Keywords:** DNA extraction, CTAB protocol, Silica matrix, Contaminant-free DNA, High-molecular-weight DNA, Next-generation sequencing, High-throughput genotyping, Recalcitrant species, SPET, Nanopore

## Abstract

**Background:**

The use of sequencing and genotyping platforms has undergone dramatic improvements, enabling the generation of a wealth of genomic information. Despite this progress, the availability of high-quality genomic DNA (gDNA) in sufficient concentrations is often a main limitation, especially for third-generation sequencing platforms. A variety of DNA extraction methods and commercial kits are available. However, many of these are costly and frequently give either low yield or low-quality DNA, inappropriate for next generation sequencing (NGS) platforms. Here, we describe a fast and inexpensive DNA extraction method (SILEX) applicable to a wide range of plant species and tissues.

**Results:**

SILEX is a high-throughput DNA extraction protocol, based on the standard CTAB method with a DNA silica matrix recovery, which allows obtaining NGS-quality high molecular weight genomic plant DNA free of inhibitory compounds. SILEX was compared with a standard CTAB extraction protocol and a common commercial extraction kit in a variety of species, including recalcitrant ones, from different families. In comparison with the other methods, SILEX yielded DNA in higher concentrations and of higher quality. Manual extraction of 48 samples can be done in 96 min by one person at a cost of 0.12 €/sample of reagents and consumables. Hundreds of tomato gDNA samples obtained with either SILEX or the commercial kit were successfully genotyped with Single Primer Enrichment Technology (SPET) with the Illumina HiSeq 2500 platform. Furthermore, DNA extracted from *Solanum elaeagnifolium* using this protocol was assessed by Pulsed-field gel electrophoresis (PFGE), obtaining a suitable size ranges for most sequencing platforms that required high-molecular-weight DNA such as Nanopore or PacBio.

**Conclusions:**

A high-throughput, fast and inexpensive DNA extraction protocol was developed and validated for a wide variety of plants and tissues. SILEX offers an easy, scalable, efficient and inexpensive way to extract DNA for various next-generation sequencing applications including SPET and Nanopore among others.

## Background

In the last decade, sequencing and genotyping technologies have become routine, allowing to generate a wealth of genomic information even in non-model plant species and neglected crops [1, 2]. Nowadays, genome sequencing, as well as the most common high-throughput genotyping strategies, like Genotyping-by-Sequencing (GBS [3]), Restriction Associated DNA Sequencing (RADseq [4]) and Single Primer Enrichment Technology (SPET [5, 6]), are conducted using next-generation sequencing (NGS) platforms. However, despite the advances, DNA quality is still a main bottleneck, mostly for the third-generation sequencing platforms where high-molecular-weight DNA free of contaminants is required [7]. Unlike bacteria and mammalian cells, fungi and plant cells are protected by rigid polysaccharide cell walls that hamper the extraction of unfragmented DNA [8]. Furthermore, plants produce a wide array of compounds and secondary metabolites (e.g., pigments, phenols, carbohydrates, waxes, among others) that tend to co-precipitate with the DNA and interfere with the subsequent enzymatic reactions [9].

So far, the CTAB DNA extraction protocol developed by Doyle and Doyle [10] is one of the most widely used by plant researchers. Several modifications of this protocol have been implemented in order to minimize contamination by other compounds of specific tissues of species [7, 11, 12]. These modifications, apart from being species or tissue-specific and frequently not removing completely interfering compounds, are time-consuming due to many handling steps, and thus are not suitable for high-throughput applications [13, 14].

Conversely, commercial kits based on silica matrices avoid many of these issues by optimizing the conditions in which only DNA can bind to the silica surface. Therefore, contaminants such as polysaccharides, polyphenols and proteins can be easily removed [15]. They also tend to be faster than the standard CTAB protocol, being the preferred option for sequencing studies in which many samples must be evaluated [9, 16]. Usually, commercial kits rely on the reversible interaction between DNA and a silica or silicate support, either in the form of a filter membrane or of silica-coated magnetic particles [17, 18]. The adsorption of DNA to the silica surface is facilitated by buffers with low pH, high concentrations of chaotropic salts (such as guanidinium hydrochloride, guanidinium thiocyanate, or sodium iodide) and ethanol [19–22]. Under these conditions, the surface of the silica can interact with the negative surface of DNA via ionic interactions [23, 24]. After several washes with high concentrations of ethanol to eliminate contaminants, DNA is generally eluted with water or TE at pH 8.0. At this higher pH value, the negatively charged silica surface and DNA repeal each other, releasing the DNA [25–27].

However, commercial kits are usually expensive, with reagent costs commonly ranging between 2 and 9 US$ per sample [8, 28], and many times provide low yields, insufficient for some NGS applications [8, 29]. Furthermore, for some commercial kits, the DNA quality and quantity obtained in recalcitrant species is low [30–32]. DNA extraction methods relying on silica matrices and chaotropic salts have been reported [33–36]; however, chaotropic salts can inhibit subsequent enzymatic reactions which are essential for NGS applications [37–39].

In this study, we present a novel, fast and inexpensive DNA extraction protocol that combines the advantages of CTAB-based extraction coupled with a purification on a silica matrix. The new method was assessed on different species, including recalcitrant ones and different tissues. To test its suitability for different NGS applications, the method was compared with commercial kits for Single Primer Enrichment Technology (SPET) genotyping [6]. The method was also used to extract high-molecular-weight DNA from a recalcitrant wild species (*Solanum elaeagnifolium*). The DNA obtained was successfully used to construct long insert size Nanopore libraries for a de novo genome assembly, which can be difficult for recalcitrant species [40], thus proving its suitability for third-generation sequencing platforms.

We demonstrate that this new method combines the advantages of commercial kits (high-quality DNA, fast and broad range of species spectrum) with those of a CTAB-based method (high yield and inexpensive) being suitable for routinely DNA screening and NGS platforms.

## Materials

### Plant material

To test our proposed protocol (hereafter named SILEX, for SILica matrix EXtraction), leaf and fruit tissue from non-recalcitrant species and leaf tissue from recalcitrant species was sampled for four different trials. In a first trial, leaf tissue from a total of 1860 accessions of tomato (*S. lycopersicum*) and its wild relatives was extracted to compare the quality, quantity and integrity of DNA extracted using SILEX and the commercial kit sbeadex maxi plant kit (hereafter SMP kit; LGC Genomics, Teddington, UK) for SPET genotyping. Extractions were performed on different days over several months.

In a second trial, in order to evaluate the appropriateness of SILEX in different plant tissues, 50 mg of fresh and 30 mg of lyophilized fruit tissue of tomato, eggplant (*S. melongena*) and pepper (*Capsicum annuum*) were extracted. The fruit tissue was collected, immediately frozen in liquid N_2_ and lyophilized In a third trial, the suitability of SILEX for DNA extraction in recalcitrant species was assessed using leaves tissue of six species, cassava (*Manihot esculenta)*, grapevine (*Vitis vinifera*), loquat (*Eriobotrya japonica*), banana (*Musa *×* paradisiaca*), naranjillo (*Solanum bonariense*), and strawberry (*Fragaria *×* ananassa*), selected to represent a wide range of recalcitrant species presenting different contaminants and secondary metabolites that interfere with DNA extraction. Extractions from recalcitrant plants made by SILEX were compared with those carried out using the standard CTAB protocol [10] and the commercial SMP kit following the manufacturer’s instructions. Finally, the suitability of SILEX to extract clean and high-molecular-weight DNA for third-generation sequencing was assessed in the silverleaf nightshade (*S. elaeagnifolium*), a wild relative of eggplant [41], that we selected for the difficulty to obtain contaminant-free DNA due to its high content in phenolics [42].

### Solutions, reagents and consumables

- 2 ml Sarstedt Microtube (https://www.sarstedt.com, Cat No. 72.691).
- 5 mm Glass beads (https://www.vwr.com, Cat No. MARI4901005).
- N-Cetyl-*N*,*N*,*N*-trimethylammonium bromide, CTAB (https://www.itwreagents.com, Cat No. A6284.0500).
- Polyvinylpyrrolidone, PVP-40 (https://www.sigmaaldrich.com, Cat No. PVP40-500G).
- Ethylenediaminetetraacetic acid, EDTA (https://www.itwreagents.com, Cat No. 131026.1210).
- Trizma^®^ hydrochloride, Tris HCl (https://www.sigmaaldrich.com, Cat No. 93363-500G).
- Sodium Chloride (https://www.itwreagents.com, Cat No. 131659.1211).
- β-Mercaptoethanol (https://www.sigmaaldrich.com, Cat No. M6250-100ML).
- RNase A (https://www.vwr.com, Cat No. NA-03).
- Chloroform Essent^®^ (https://www.scharlab.com, Cat No. CL1981000).
- Isoamyl alcohol Essent^®^ (https://www.scharlab.com, Cat No. AL02851000).
- Polyethylene Glycol 8000 (https://www.itwreagents.com, Cat No. 146224.1211).
- Absolute Ethanol ExpertQ^®^ (https://www.scharlab.com, Cat No. ET0002025P).
- Silicon dioxide (https://www.fishersci.se, Cat No. S5631-500G).
- Hydrochloric acid (https://www.sigmaaldrich.com, Cat No. H1758-500ML).
- Absolute Ethanol ExpertQ^®^ (https://www.scharlab.com, Cat No. ET0002025P).
- Tris Base (https://www.itwreagents.com, Cat No. 14194.1211).

### Equipment

- Qiagen TissueLyser II (https://www.qiagen.com, Cat No. 85300).
- Thermoblock (https://www.vwr.com, Cat No. 12621-096).
- Eppendorf Centrifuge 5424 (https://www.eppendorf.com, Cat. No. 5404000010).

### Reagent setup

- Extraction buffer: 2% (w/v) CTAB, 2% (w/v) PVP-40, 20 mM EDTA, 100 mM Tris HCl (pH 8.0) and 1.40 M NaCl. The buffer may be stored for several months at room temperature.
- Protein precipitation buffer: 24 parts of chloroform and 1 part of isoamyl alcohol. It may be stored for several months at room temperature.
- Binding buffer: 2.5 M NaCl and 20% PEG 8000. It may be stored for several months at room temperature.
- Silica matrix buffer: Mix 5 g of silicon dioxide with 50 ml of MilliQ water and let stand for 24 h. Discard the supernatant and resuspend the pellet in 50 ml of MilliQ water and let stand for another 5 h. Discard the supernatant and resuspend the pellet in 1:1 (v/v) MilliQ water. Finally, add 10 µl of HCl 36% per ml of silica matrix solution obtained. It may be stored for several months at room temperature.
- Washing buffer: Fresh prepared ethanol 70%. It may be stored for several months at room temperature.
- Elution buffer: 10 mM Tris HCl (pH 8.0) and 1 mM EDTA (pH 8.0). It may be stored for several months at room temperature.

### Protocol

#### SILEX DNA extraction protocol

1. Add a 5 mm glass bead to a 2 ml tube, place around 50 mg of fresh or lyophilized tissue into the tube and immediately frozen in liquid nitrogen.
2. Place the tubes in the Qiagen TissueLyser adapters, grind the samples for 60 secs at 13,000 rpm and immediately return the samples in liquid nitrogen.
  **NOTE:** This is a critical step and can greatly influences the final DNA recovery. Avoid sample thaw and pre-chill Qiagen TissueLyser plate adapters. Check that the plant material has become a fine powder.
3. Take the tube from the liquid nitrogen and gently tap it vertically to settle the plant material at the bottom of the tube. Add 1 ml of extraction buffer, add 14 µl of β-mercaptoethanol and gently mix the tube until complete homogenization. Then, add 2 µl of RNase (10 mg/ml) and incubate in a thermoblock for 30 min at 65 °C.
  **NOTE:** Avoid sample thaw before adding the extraction buffer. Preheat the thermoblock at 65 °C.
4. Put the samples on ice for 5 min. Add 700 µl of protein precipitation buffer and gently vortex or (for very high molecular weight DNA) gently invert by hand thoroughly for approximately 20 s.
5. Centrifuge at 11,000 rpm for 5 min at room temperature, carefully recover around 800 µl of the supernatant phase and transfer it to a new 2 ml tube.
  **NOTE:** Pipette carefully to avoid dragging the interphase. Interphase contaminants can largely affect the final quality of the DNA.
6. Add 480 μl of binding buffer and gently invert the tube by hand until complete mixing. Subsequently, add 720 μl of absolute ethanol and gently invert the tube again for a few seconds until complete mixing.
  **NOTE:** The amount of binding buffer plus ethanol must be 1.5 volumes of the supernatant recovered in step 5. In the binding buffer plus ethanol mix, 40% should be the binding buffer and 60% should be absolute ethanol.
7. Add 20 µl of silica matrix buffer and mix gently during 5 min (by hand or using an orbital shaker).
8. Spin down the silica for 5 to 6 s and discard the supernatant by decantation.
  **NOTE:** Longer centrifugation times will make it difficult to resuspend the silica in the subsequent steps.
9. Add 700 μl of washing buffer and shake gently by hand until a uniform dispersion of the silica is obtained.
10. Spin down the silica for 5 to 6 s, gently discard the supernatant by decantation and let dry at room temperature for 5 min.
  **NOTE:** Make sure that all ethanol is completely evaporated.
11. Add 100 µl of elution buffer, shake gently by hand until the pellet is resuspended and incubate 5 min at 65 °C.
12. Centrifuge at 14,000 rpm for 10 min at room temperature and transfer 90 µl of the supernatant to a new tube.

#### DNA concentration and quality

DNA integrity was checked by electrophoresis on a 0.8% agarose gel (Condalab, Madrid, Spain) in 1X TAE buffer (GenoChem World, Valencia, Spain) stained with GelRed^®^ (Biotium, Fremont, CA, USA) at a constant voltage of 100 V for 50 min. Gel Doc XR + System transilluminator (Bio-Rad, Hercules, CA, USA) was used to visualized agarose gels. For high-molecular-weight DNA, the size and integrity were tested by pulse-field gel electrophoresis was run at 3.3 V/cm in 15-second cycles with an angle of 120º for 24 h at 4 °C with 0.8% agarose in TB buffer.

DNA yield and quality were measured spectrophotometrically using NanoDrop™ ND-1000 (Thermo Scientific, Waltham, MA, USA). A_260_/A_280_ and A_260_/A_230_ ratios were measured to determine, respectively, protein and polysaccharide contamination. DNA quantity was also quantified with a Qubit™ 2.0. Fluorometer (Thermo Scientific, Waltham, MA, USA). An aliquot of 2 µl of each sample was examined using the Qubit™ dsDNA BR Assay Kit (Thermo Scientific, Waltham, MA, USA) according to the instructions of the manufacturer.

In addition, the concentration of DNA obtained from the 1860 tomato samples was measured fluorometrically using Quant-iT™ PicoGreen™ dsDNA Assay Kit (hereafter PicoGreen, Thermo Scientific, Waltham, MA, USA) and a 96-wells plate reader VICTOR3 1420 (PerkinElmer, Waltham, MA, USA) equipped with an excitation filter F485 and emission filter F535.

To check the suitability of the DNA extraction method for sequencing applications where DNA is fragmented, approximately 1 µg of DNA was digested for 1 h at 37 °C followed by 20 min at 65 °C with restriction enzymes *Eco*RI (New England Biolabs, Ipswich, MA, USA). The digestion was evaluated through 1% agarose electrophoresis as above.

#### High-throughput genotyping quality check

For the first trial, sequencing of tomato samples for genotyping by SPET was performed with an Illumina NextSeq500 platform (Illumina Inc., San Diego, CA, USA), following the manufacturer protocol. *Phred* values were obtained using FastQC Version 0.11.8. and plotted in R [43] using the package ggplot2 [44].

#### Suitability of extracted DNA for third-generation sequencing platforms

For the third trial, 5 µg of *S. elaeagnifolium* DNA from a single extraction were size-selected using the Circulomics SRE-XL-Kit (Circulomics Inc., Baltimore, MD, USA). For library preparation, 1 µg of the size-selected DNA was used to prepare each of the three Nanopore LSK-109 libraries. Two of these libraries were sequenced on a MinION R9.4.1 (Oxford Nanopore, Oxford, UK) and the third was loaded on a PromethION PRO-002 (Oxford Nanopore, Oxford, UK). All three sequencing runs were basecalled using Oxford Nanopores Guppy basecaller version 3.2.2 (Oxford Nanopore, Oxford, UK) using the high accuracy basecalling models.

## Results

### Comparison of DNA extraction methods

#### Tomato leaf samples

Total DNA yield extracted through the SMP kit and estimated by NanoDrop ranged from 14.5 ng/mg to 366.9 ng/mg with a mean of 38.3 ng/mg and a standard deviation (SD) of 29.2 ng/mg. DNA extracted by SILEX showed higher output, ranging from 86.1 ng/mg to 1698.1 ng/mg with an average of 382.9 ng/mg and a SD of 205.3 ng/mg (Table 1). Despite higher SD, the coefficient of variation (CV) of SILEX (53.6%) was lower than that of the SMP kit (76.1%).

**Table 1:** Mean value, standard deviation (SD), range and coefficient of variation (CV) of the DNA yield (ng/mg) using SILEX and SMP kit and quantified by NanoDrop (ND) and PicoGreen (PG)

	SILEX		SMP kit	
	ND	PG	ND	PG
Samples (n)	1380		480	
Mean	382.9	141.3	38.3	41.7
SD	205.3	36.8	29.2	26.4
Range	86.1–1698.1	37.9–231.2	14.5–366.9	1.2–134.8
CV (%)	53.6	26.1	76.1	63.4
Ratio ND/PG	2.7		0.9	

**Fig. 1:**
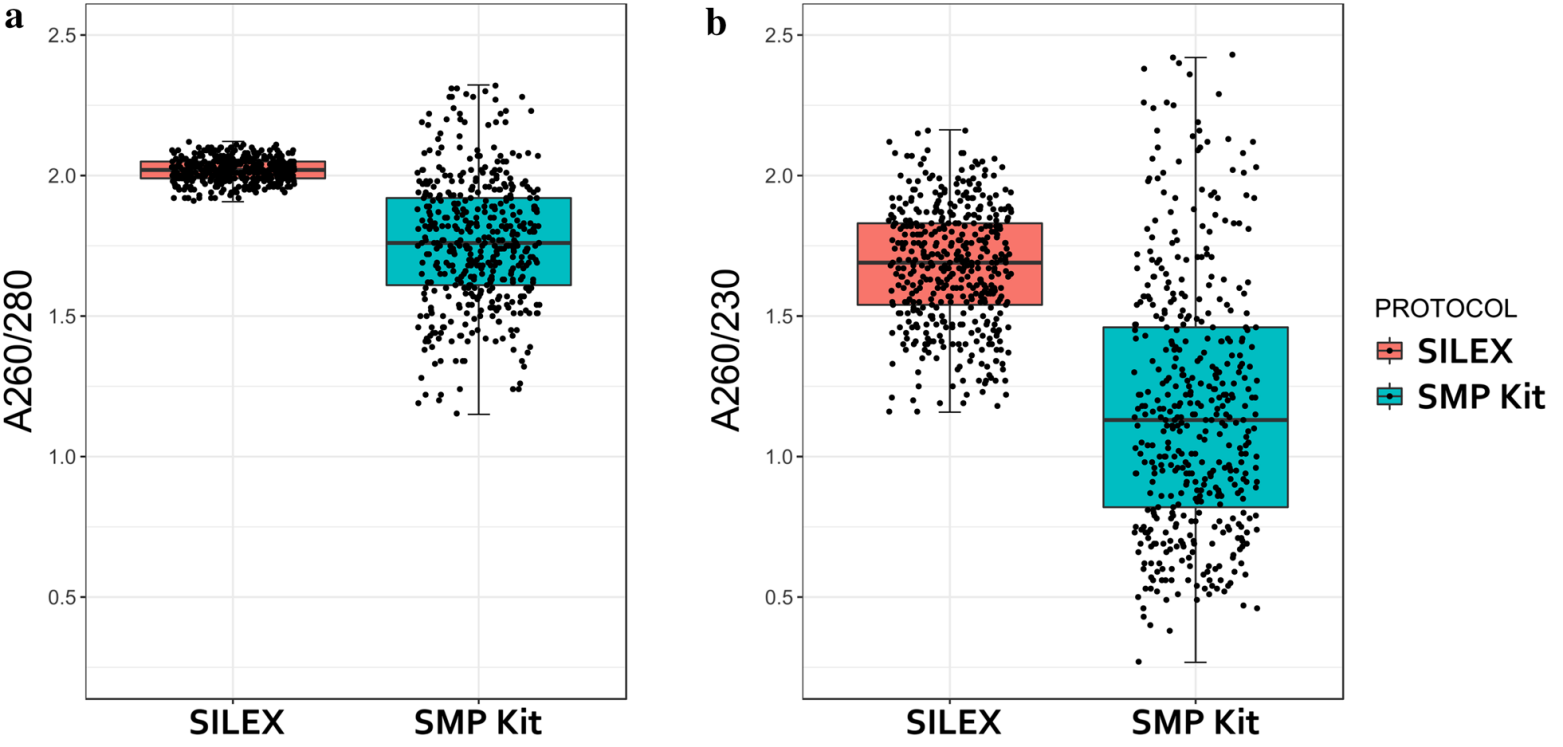
Quality control of DNA extracted with SILEX (orange) and SMP kit (blue) in 1380 and 480 tomato samples respectively. Box and whisker plots are based on **a** A_260_/A_280_ and **b** A_260_/A_230_. Each dot represents a sample, the median is indicated by a horizontal line, the box represents the upper and lower quartiles and the whiskers show the variability outside the quartiles.”

The A_260_/A_280_ ratio, which indicates protein contamination, was very variable in the SMP kit protocol, ranging from 1.15 to 2.32, with an average of 1.76 and a SD of 0.33 (Fig. 1a). In contrast, SILEX showed a more consistent ratio with less variation (from 1.91 to 2.12) and with an average value of 2.03 and a SD of 0.05. Similarly, for the A_260_/A_230_ ratio, which indicates salt and carbohydrates contamination, SMP kit showed a greater dispersion, with a ratio between 0.27 and 2.43 with an average of 1.09 and a SD of 2.55, compared to SILEX, which ranged from 1.16 to 2.16 with an average of 1.66 and a SD of 0.25 (Fig. 1b) (Table 2).

**Table 2:** Mean value, standard deviation (SD), range and coefficient of variation (CV) of NanoDrop absorbance ratios (A_260_/A_280_ and A_260_/A_230_) using SILEX and SMP kit

	SILEX		SMP kit	
	A_260_/A_280_	A_260_/A_230_	A_260_/A_280_	A_260_/A_230_
N samples	1380		480	
Mean	2.03	1.66	1.76	1.17
SD	0.05	0.22	0.22	0.45
Range	1.91–2.12	1.16–2.16	1.15–2.32	0.27–2.43
CV (%)	2.5	13.3	12.5	38.5

N samples indicate the number of independent extractions performed

Since spectrophotometric measurements with NanoDrop tend to overestimate DNA yield due to likely interferences of proteins [45], those measures were compared with the fluorometric ones performed with PicoGreen. Yields estimated by the latter ranged from 1.2 ng/mg to 134.8 ng/mg with a mean of 41.7 ng/mg and a SD of 26.4 ng/mg in the case of DNA extracted by SMP kit. On the other hand, SILEX had higher yields, ranging from 37.9 ng/mg to 231.2 ng/mg with a mean of 141.3 ng/mg and a SD of 36.8 ng/mg (Table 1). In addition, yields estimated by PicoGreen had greater variation between samples in DNA extracted by SMP kit (63.4%) in comparison with that extracted by SILEX (26.1%).

To assess the overestimation of DNA yield extracted using the different protocols, we compared the ratios obtained by NanoDrop and PicoGreen measurements. Estimation of yield by NanoDrop of DNA extracted with the SMP kit showed an estimation of 0.9-fold compared to PicoGreen, suggesting that for this commercial kit NanoDrop measurements were comparable with the PicoGreen ones. In contrast, NanoDrop measurements from SILEX tended to overestimate DNA yield 2.7-fold compared to PicoGreen, suggesting contamination with a molecule absorbing at 260 nm. One possible explanation for this overestimation is that remnants of degraded RNA were present in our samples, since nanodrop is unable to discriminate among free nucleotides, RNA, single-stranded DNA, and double-stranded DNA. However, even with this overestimation, the average yield obtained with SILEX (141.3 ng/mg with PicoGreen) was 3.4 times higher than with SMP kit (41.7 ng/mg).

#### DNA extraction from dry and fresh fruit tissues

The amount of DNA obtained from dry and fresh fruit tissues was similar to that achieved using leaf tissue and ranged from 116.4 to 920.3 ng/mg. In general, higher yield of DNA was obtained with lyophilized tissue (Table 3). Regardless of the tissue used, A_260_/A_280_ ratios were above 2.0 which indicates no protein contamination. On the other hand, lower ratios were observed in A_260_/A_230_ ratio, suggesting the presence of some organic contaminants. Despite these ratios, DNA obtained was successfully digested by *Hind*III restriction enzyme.

**Table 3:** Yield and quality control of DNA extracted by lyophilized and fresh fruit tissue of tomato, eggplant and pepper measured using NanoDrop. Mean value and standard deviation of 12 independent extractions are shown

Species	Fruit sample	Yield (ng/mg)	Absorbance ratios	
			A_260_/A_280_	A_260_/A_230_
Tomato	Lyophilized	116.4 ± 65.3	2.08 ± 0.08	1.30 ± 0.11
	Fresh	237.6 ± 40.2	2.22 ± 0.04	0.98 ± 0.08
Eggplant	Lyophilized	863.0 ± 289.0	2.10 ± 0.05	1.36 ± 0.13
	Fresh	253.0 ± 63.8	2.11 ± 0.04	1.38 ± 0.06
Pepper	Lyophilized	920.3 ± 248.7	2.10 ± 0.04	1.46 ± 0.14
	Fresh	271.6 ± 54.0	2.08 ± 0.03	1.18 ± 0.15

Yield and quality control of DNA extracted by lyophilized and fresh fruit tissue of tomato, eggplant and pepper measured using NanoDrop.

#### DNA extraction from recalcitrant species

Overall, SILEX resulted in higher DNA yields, ranging (with fluorimetric determination) from 46.4 ng/mg in strawberry to 318.0 ng/mg in grapevine, than those obtained with the standard CTAB method or the SMP kit (Table 4). In addition, A_260_/A_280_ ratios obtained with SILEX were above 2.0, which is considered a protein-free DNA, except in strawberry where values were on average 1.86, even though they were higher than in standard CTAB and SMP kit. In the same way, SILEX A_260_/A_230_ ratios were higher than in the two other protocols, from 1.71 in loquat to 2.16 in banana, except in strawberry, where the results were similar to standard CTAB and SMP kit (Table 4). The differences were very noticeable in banana and grapevine, where the A_260_/A_230_ ratios were 2.7 and 4.3-fold lower for standard CTAB and 1.7 and 3.3-fold lower for SMP kit, respectively (Table 4). NanoDrop/Qubit ratio estimated with SILEX for recalcitrant species seemed to be species-dependent, and ranged from 1.4-fold in grapevine to 6.6-fold in naranjillo with a mean of 3.9-fold in comparison to Qubit. However, even though the SMP kit provided lower NanoDrop/Qubit ratios, SILEX performed better than the standard CTAB, which on average had a NanoDrop/Qubit ratio of 18-fold.

**Table 4:** Yield and quality control of DNA extracted from six recalcitrant species using three different methods: SILEX, standard CTAB, and SMP kit quantified by NanoDrop (ND) and Qubit (Q)

Species/Methods	Yield (ng/mg)		Absorbance ratios		Ratio ND/Q
	ND	Q	A_260_/A_280_	A_260_/A_230_	
Cassava					
SILEX	556.0 ± 76.6	106.9 ± 17.4	2.04 ± 0.02	2.13 ± 0.05	5.3
CTAB	512.7 ± 61.1	37.7 ± 27.7	2.12 ± 0.02	1.96 ± 009	13.6
SMP kit	75.4 ± 9.4	85.5 ± 13.9	1.80 ± 0.09	1.44 ± 0.10	0.9
Grapevine					
SILEX	442.8 ± 24.1	318.0 ± 115.2	2.07 ± 0.02	1.93 ± 0.01	1.4
CTAB	394.3 ± 18.7	21.5 ± 6.1	1.60 ± 0.05	0.44 ± 0.03	18.4
SMP kit	50.7 ± 30.2	0.0 ± 0.0	1.73 ± 0.20	1.09 ± 0.09	∞
Loquat					
SILEX	284.4 ± 24.9	92.7 ± 35.5	2.02 ± 0.05	1.71 ± 0.06	3.1
CTAB	112.7 ± 7.5	4.3 ± 2.1	1.58 ± 0.17	0.57 ± 0.08	25.9
SMP kit	66.7 ± 36.0	7.7 ± 6.1	1.78 ± 0.25	1.15 ± 0.41	8.6
Banana					
SILEX	267.8 ± 5.1	59.2 ± 8.2	2.13 ± 0.01	2.16 ± 0.05	4.5
CTAB	202.0 ± 37.5	19.1 ± 5.6	2.20 ± 0.14	0.78 ± 0.13	10.6
SMP kit	31.4 ± 37.5	1.3 ± 2.3	1.47 ± 0.31	0.74 ± 0.32	23.3
Naranjillo					
SILEX	1184.1 ± 484.3	180.4 ± 60.6	2.06 ± 0.02	1.85 ± 0.03	6,6
CTAB	525.4 ± 126.2	26.0 ± 7.7	1.91 ± 0.11	0.86 ± 0.20	20.2
SMP kit	105.4 ± 35.8	42.6 ± 19.8	1.82 ± 0.04	1.72 ± 0.07	2.5
Strawberry					
SILEX	193.8 ± 5.1	46.4 ± 6.1	1.86 ± 0.05	1.02 ± 0.08	4.2
CTAB	405.3 ± 108.5	24.6 ± 4.0	1.77 ± 0.08	1.14 ± 0.11	16.5
SMP kit	25.9 ± 2.8	20.0 ± 14.3	1.69 ± 0.16	0.97 ± 0.02	1.3

Ratios of the latter are reported. Mean value and standard deviation are based on a minimum of three independent extractions

**Fig. 2:**
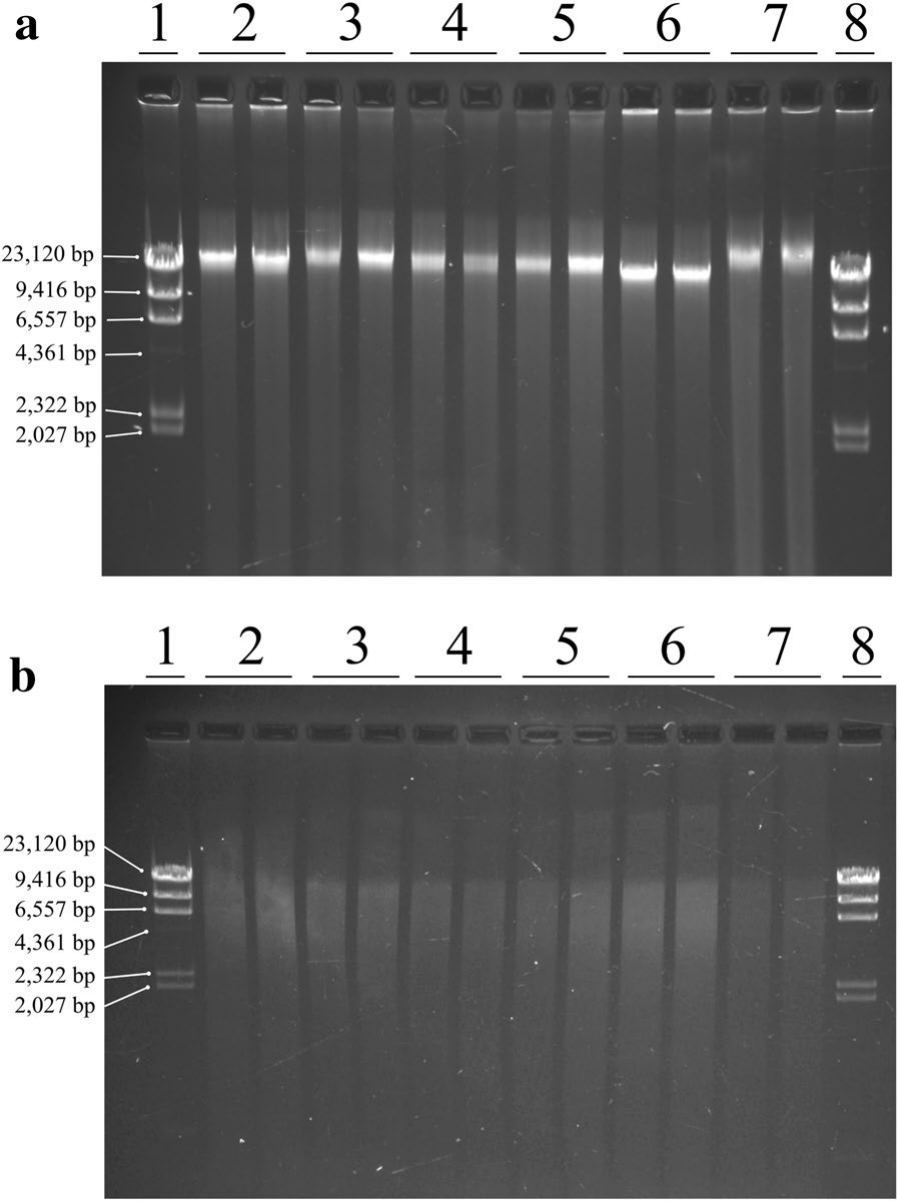
Agarose gel electrophoresis of uncut genomic DNA extracted from six recalcitrant species with SILEX (**a**) and the same DNA cut with *Eco*RI enzyme (**b**). Two biological replicates for each species are shown. *Lambda* DNA restricted with *Hind*III (lane 1 and 8); cassava (lane 2); grapevine (lane 3); loquat (lane 4); banana (lane 5); naranjillo (lane 6) and strawberry (lane 7)

In order to test if the presence of contaminants could inhibit the enzyme activity, DNA was digested with the restriction enzyme *Eco*RI. Agarose gels, such as the one shown in Fig. 2 indicated efficient endonuclease activity in all the DNA extracted from the six recalcitrant species even though in some cases A_260_/A_230_ ratios were below 1.8 (strawberry and loquat). Also, strawberry samples showed yellow and brown coloration and high viscosity even after two washing steps.

### SILEX timing and cost

**Fig. 3:**
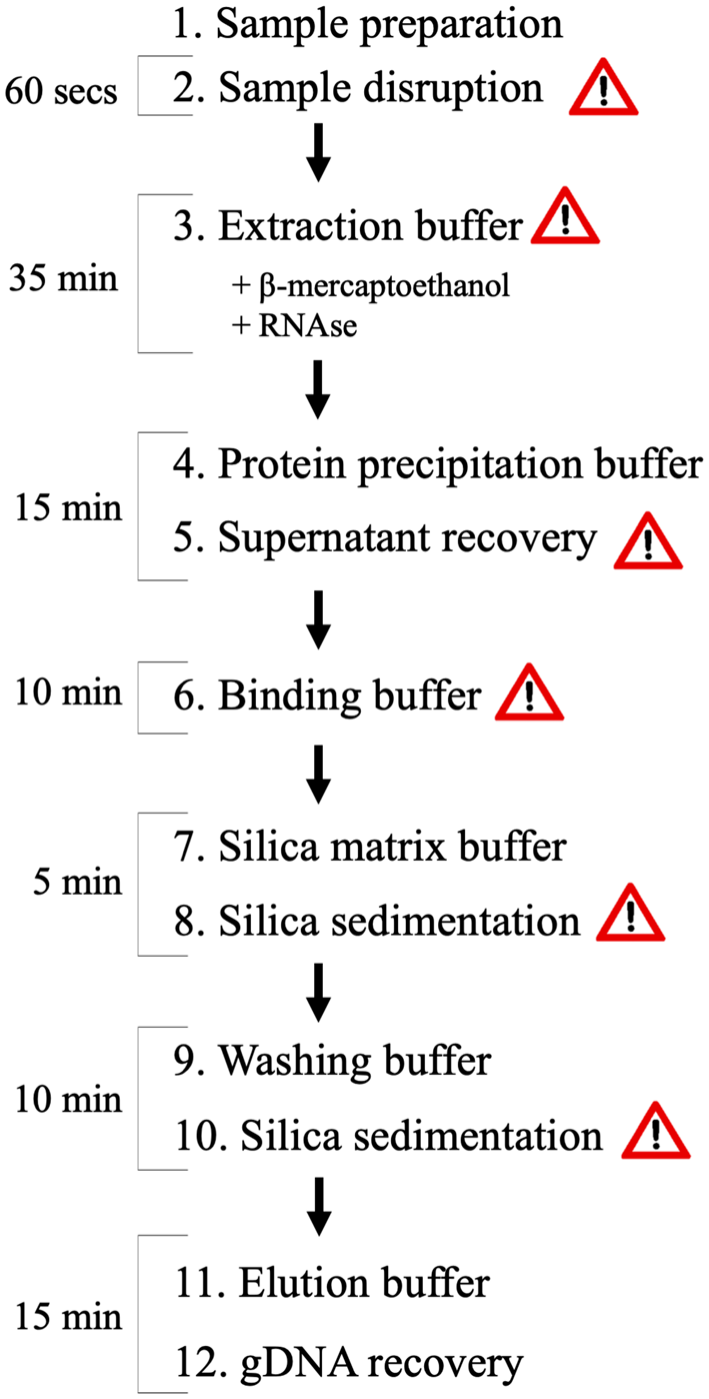
Flowchart of the twelve steps of SILEX DNA extraction method and timing estimation to perform 48 samples in tubes. The warning signal indicates key points (see in the text the notes in the protocol section)

The time needed to extract 48 samples, without taking into account the sampling of the plant material) is approximately 96 min (around 2 min/sample; Fig. 3). The estimated cost of all consumables required to extract high-molecular-weight gDNA using SILEX is approximately 0.12 € per sample (Additional file 1: Additional data S1).

### High-throughput genotyping platforms

**Fig. 4:**
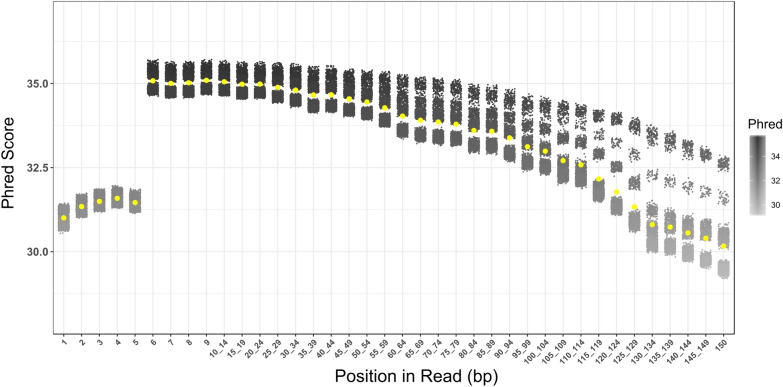
Summary of *Phred* values for 1380 tomato samples extracted with the SILEX protocol and genotyped with SPET along the 150 bp sequence. Each dot corresponds to one sample. Yellow spots indicate the mean value

In order to evaluate the suitability of the gDNA obtained with SILEX for high-throughput genotyping, 1380 tomato samples were genotyped using SPET [6]. The reads obtained showed excellent *Phred*-quality scores along the 150 bp, with a mean value of 33.1 (Fig. 4). Similar results were obtained with 480 samples extracted using the SMP kit with a mean value of 33.5. The mean *Phred* score along the 150 bp sequenced was always over 30, indicating good sequencing quality in both methods, with the SILEX method providing more DNA per equal amount of tissue.

### High molecular weight DNA extraction

**Fig. 5:**
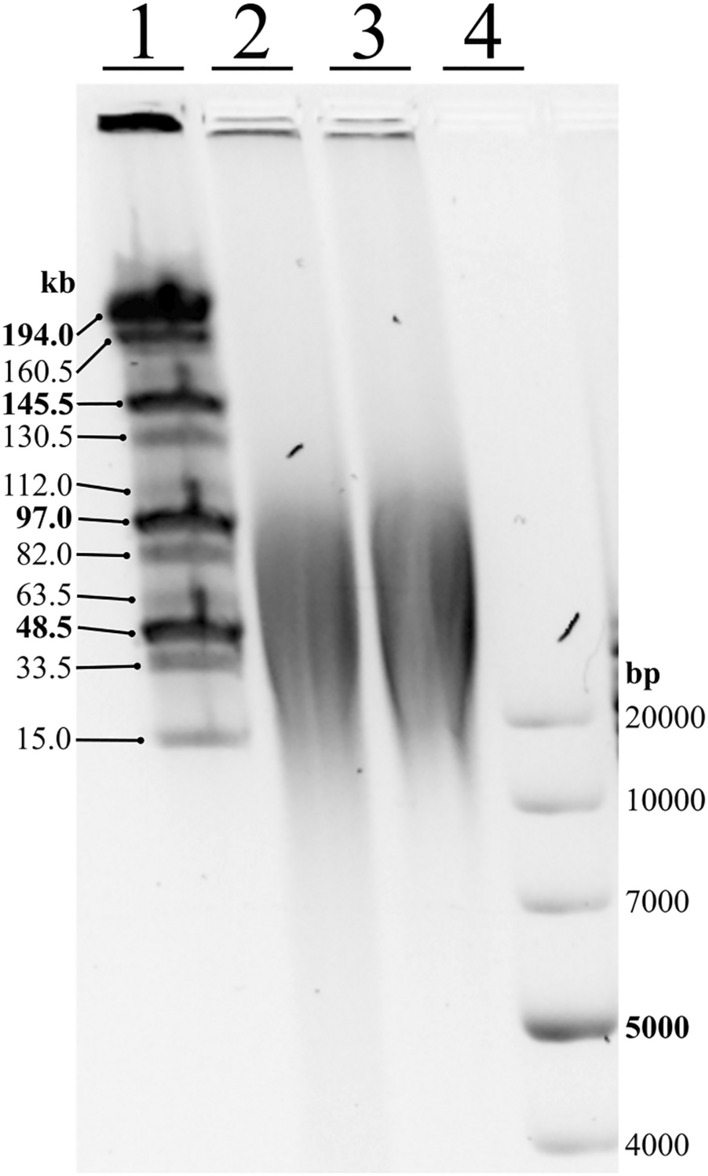
*Solanum elaeagnifolium* gDNA size estimation using PFGE after extraction with SILEX. Line 1 is MidRange PFG Marker (New England Biolabs, Ipswich, USA) and line 4 is GeneRuler 1 kb Plus DNA (Thermo Fisher Scientific, Waltham, USA). Line 2 shows the gDNA before size selection and line 3 the gDNA before size selection after using Short Read Eliminator XL Kit

To test the suitability of using SILEX for NGS platforms requiring high-molecular-weight DNA, *S. elaeagnifolium* DNA was size-selected using the Circulomics short read eliminator kit, recovering 3.5 µg, and analysed using Pulsed-field gel electrophoresis (PFGE) (Fig. 5). The size-selected DNA ranged from 20 to 100 Kb and contained relatively little small fragments. The sizes obtained were suitable for most sequencing platforms that require high molecular weight DNA free of impurities such as Nanopore or PacBio. Two libraries were sequenced with a MinION sequencer and yielded 6.8 and 7.5 Gbp, with N50 values of 22.8 and 28.2 kbp, respectively, while the third library, sequenced with a PromethION sequencer, resulted in 55.7 Gbp of raw data with N50 of 24.2 kbp. After the base-calling, the total sequencing yield was 70.1 Gbp.

## Discussion

One of the main advantages of the SILEX protocol for DNA extraction is the use of common and inexpensive reagents and its simplicity. No toxic salts such as guanidinium thiocyanate or sodium iodide at high concentrations are used. Several authors reported that the use of NaCl at concentrations higher than 2 M facilitated the DNA binding to the silica surface [46–48]. Also, it is known that the addition of polyethylene glycol (PEG) to the binding solution increases the adsorption due to the compact globular structure of DNA adopted under these conditions [49]. For these reasons, we use a binding buffer composed by the non-toxic, inexpensive NaCl and PEG compound to facilitate the DNA binding to the silica surface. The total cost of reagents and consumables is only 0.12 € per sample and for multiple simultaneous manual extractions, each sample requires less than 2 min per person. In this respect, in the SILEX method, the silica matrix used for each extraction cost less than 0.001 € and the washing buffer is only water and ethanol, a common non-toxic reagent in most molecular biology laboratories.

The protocol presented here has been tested on many samples of different species with similar satisfactory results, confirming its wide applicability. The quality and quantity parameters obtained also indicate that SILEX is at least as effective as commercial kits even when recalcitrant species were used. In recalcitrant species, the presence of polysaccharides and phenols was significantly lower in SILEX compared to the standard CTAB protocol where several samples showed yellow and brown coloration and high viscosity, indicating the presence of oxidized polyphenols and high concentration of polysaccharides. One of the reasons for this difference could be the absence of a precipitation step in SILEX, as polysaccharides and polyphenols tend to co-precipitate with DNA when isopropanol or ethanol is added [50]. This is important since the presence of polysaccharides such as carrageenan, pectin and xylan are strong inhibitors of PCR [51, 52]. We also observed that in species with very high polyphenol and polysaccharide compounds, such as strawberry [53], a second washing step increases the A_260_/A_230_ ratio. Our DNA extraction protocol has been tested by other research groups and it has been found to provide high-quality DNA in high concentrations in other plant species as different as silver fir (*Abies alba*), watermelon (*Citrullus lanatus*), melon (*Cucumis melo*), summer squash (*Cucumis pepo*), common fig (*Ficus carica*), lettuce (*Lactuca sativa*), European larch (*Larix decidua*), Spanish stonecrop (*Sedum hispanicum*), avocado (*Persea americana*) and sweet cherry (*Prunus avium*). Applications have included genotyping by SSRs (Single Sequence Repeats), HRM (High-Resolution Melting), and GBS among others.

Although DNA is usually extracted from fresh leaf tissue, it is sometimes necessary to use other types of material such as fresh or freeze-dried fruit. Our protocol was flexible enough to successfully extract high DNA quantities from lyophilized and fresh fruit tissues obtaining A_260_/A_280_ ratios above 2.0.

Thousands of samples of tomato and wild relatives were successfully genotyped using SPET high-throughput genotyping, that relays on DNA fragmentation, target probe annealing, PCR amplification and NGS sequencing [6]. The quality of the reads produced had a mean *Phred* value over 30, which represents a base call accuracy of 99.9%. Also, hundreds of samples of grapevine and watermelon were genotyped using GBS, obtaining similar results (C. Esteras, personal communication). This indicates the suitability of SILEX to yield DNA of enough quality to be used in different genotyping platforms. Furthermore, our DNA extraction method could be used in applications requiring high molecular weight genomic DNA, such as long-read single molecule Nanopore sequencing [54] without any additional steps.

## Conclusions

The SILEX protocol presented here is very robust and can be used in a wide variety of plants (including recalcitrant ones) and several tissues. It is based on common reagents without the need of expensive salts or equipment. This makes it inexpensive (0.12 € of reagents and consumables per sample) and accessible to most laboratories. It is also a fast method, where a trained person could process up to 48 samples in 96 min using Eppendorf tubes or 192 min if the extraction is performed in 96-well plates. The protocol is also amenable to automatization specially in labs that already have automatic DNA extraction robots. This could save hands-on time and increase the number of samples processed per day. We demonstrate that this new method gathers the advantages of commercial kits (high-quality DNA, fast and broad species spectrum) with those of the CTAB-based method (high yield and inexpensive), being suitable for routine DNA extraction for multiple applications, including NGS platforms.

## Supplementary information

**Additional file 1.** Detailed cost per sample extracted for all solutions, reagents and consumables used for SILEX workflow.

## Data Availability

The datasets used and/or analysed during the current study are available from the corresponding author on reasonable request.
